# A rapid and efficient platform for antiviral crRNA screening using CRISPR-Cas13a-based nucleic acid detection

**DOI:** 10.3389/fimmu.2023.1116230

**Published:** 2023-05-09

**Authors:** Lan Yang, Youcui Zhang, Wenyanbo Yi, Xue Dong, Mengwei Niu, Yingjie Song, Yao Han, Hao Li, Yansong Sun

**Affiliations:** ^1^ State Key Laboratory of Pathogens and Biosafety, Beijing Institute of Microbiology and Epidemiology, Beijing, China; ^2^ College of Life Sciences, Fujian Agriculture and Forestry University, Fujian, China

**Keywords:** CRISPR/Cas13a, crRNA screening, antiviral, nucleic acid detection, influenza A(H1N1)

## Abstract

**Introduction:**

Rapid and high-throughput screening of antiviral clustered regularly interspaced short palindromic repeat (CRISPR) RNAs (crRNAs) is urgently required for the CRISPR-Cas13a antiviral system. Based on the same principle, we established an efficient screening platform for antiviral crRNA through CRISPR-Cas13a nucleic acid detection.

**Method:**

In this study, crRNAs targeting PA, PB1, NP, and PB2 of the influenza A virus (H1N1) were screened using CRISPR-Cas13a nucleic acid detection, and their antiviral effects were confirmed by reverse transcription-quantitative polymerase chain reaction (RT-qPCR). The RNA secondary structures were predicted by bioinformatics methods.

**Results:**

The results showed that crRNAs screened by CRISPR-Cas13a nucleic acid detection could effectively inhibit viral RNA in mammalian cells. Besides, we found that this platform for antiviral crRNA screening was more accurate than RNA secondary structure prediction. In addition, we validated the feasibility of the platform by screening crRNAs targeting NS of the influenza A virus (H1N1).

**Discussion:**

This study provides a new approach for screening antiviral crRNAs and contributes to the rapid advancement of the CRISPR-Cas13a antiviral system.

## Introduction

The influenza virus is a negative-sense single-stranded RNA virus that belongs to the Orthomyxoviridae family and causes respiratory diseases (1). The worldwide influenza pandemic is particularly serious and causes hundreds of thousands of deaths worldwide annually (2). Among the influenza viruses, the influenza A virus (IAV) is highly mutated, has numerous subtypes and can cross the animal-human host species barrier, causing several influenza pandemics and posing a serious threat to human health (3–5). Currently, the treatment options for influenza are limited and mainly rely on antiviral drugs (6, 7). As a result of antigenic drift or shift, frequent changes occurring in influenza viruses and drug-resistant infections are increasing annually, presenting a serious challenge to the development of anti-influenza drugs (8, 9).

The clustered regularly interspaced short palindromic repeat (CRISPR)-associated (Cas) system is a defense mechanism that protects against viruses and plasmids in bacteria and archaea (10). Previous reports have demonstrated that CRISPR-Cas13a systems can target RNA and have been applied in nucleic acid detection, RNA editing, and antiviral applications (11, 12). The Cas13 can cleavage target RNAs (cis-cleavage) and also exhibits non-specific collateral cleavage activity (trans-cleavage) of the surrounding non-target RNAs (11, 13, 14). Previous studies reported that two main factors affect the RNase activity of Cas13a. One is the match between crRNA and target sequence, including GC content, mismatch, etc.; another factor is the formation of RNA stem-loop structure (11, 14). In terms of antiviral properties, CRISPR-Cas13 technology has been used to efficiently inhibit a variety of RNA viruses in cells and bodies, it not only has the advantages of high efficiency and low off-target effects but can also be programmed, which allows for simultaneous targeting of multiple virus subtypes to prevent the escape of drug-resistant strains (15). However, as a new antiviral technology, the screening of antiviral targets relies mainly on cell experiments currently, which are cumbersome, time-consuming, and costly. The cycles of antiviral development against new pathogens will be lengthy, limiting the application and development of antiviral technologies. Therefore, rapid and effective antiviral target screening methods are urgently required in the application of the CRISPR-Cas13 system.

Similar to intracellular antiviruses, the CRISPR-Cas13 system has also been used for nucleic acid detection (12, 16, 17). Both applications depend on the ability of crRNAs to specifically bind the Cas13 protein and cleave the target nucleic acid to activate the RNA enzyme activity of Cas13 (11, 18). As the CRISPR-Cas13 system has been developed as a highly sensitive, highly specific, and high-throughput nucleic acid detection technology *in vitro*, rapid screening of efficient antiviral targets is possible. In this study, an efficient screening technology for antivirus crRNA targets was established based on CRISPR-Cas13 nucleic acid detection using the influenza virus (H1N1) as an example. The screened crRNAs have high anti-influenza virus activity. It provides a new strategy for programmable antiviral target screening based on the CRISPR-Cas13a system.

## Materials and methods

### Design and preparation of crRNAs

An IAV (H1N1) subtype-specific crRNA was designed (**Table 1**). First, polymerase acidic protein (PA), polymerase basic protein 1 (PB1), nucleoprotein (NP), polymerase basic protein 2 (PB2), and non-structural protein (NS) sequences of influenza A virus PR8 strain were aligned using MEGA7. A nucleic acid fragment of 28 bases was designed as a crRNA. The crRNA was prepared as in the previous study (19). The product was transcripted using the HiScribe T7 Fast High Yield RNA Synthesis Kit (NEB) and incubated overnight at 37°C. Finally, crRNA was purified using Beckman Coulter RNA clean XP (Beckman Coulter, USA) at a ratio of 1:1.8.

**Table 1:** crRNA sequences in our study. The guide sequences are marked in italics.

Gene name	CrRNA name	RNA Sequence (5’→3’)
PA	PA-1	GAUUUAGACUACCCCAAAAACGAAGGGGACUAAAAC *GGUUCGAAUCCAUCCACAUAGGCUCUAA*
	PA-2	GAUUUAGACUACCCCAAAAACGAAGGGGACUAAAAC *UCAUCGGAUUGAAGCAUUGUCGCACAAA*
	PA-3	GAUUUAGACUACCCCAAAAACGAAGGGGACUAAAAC *AUCUCUUCCCUCGAUUAUUUCAAAUCUG*
	PA-4	GAUUUAGACUACCCCAAAAACGAAGGGGACUAAAAC *GCAUAUUGCUGCAAAUUUGUUUGUUUCG*
	PA-5	GAUUUAGACUACCCCAAAAACGAAGGGGACUAAAAC *AAAUCUGAAUACAUGAAGCAUACUUCCA*
	PA-6	GAUUUAGACUACCCCAAAAACGAAGGGGACUAAAAC *UCAUACAAAUCUGGUAGAAACUUUGGUU*
PB1	PB1-1	GAUUUAGACUACCCCAAAAACGAAGGGGACUAAAAC *GGAAAGCCAUUGCUUCCAAUACACAAUC*
	PB1-2	GAUUUAGACUACCCCAAAAACGAAGGGGACUAAAAC *CCUGGGGUUGCAAUUGCUCUCCGUUUUA*
	PB1-3	GAUUUAGACUACCCCAAAAACGAAGGGGACUAAAAC *UAUACUCCUUGCCAGUGUCUCAACAAAG*
	PB1-4	GAUUUAGACUACCCCAAAAACGAAGGGGACUAAAAC *AUCCAUGGUGUAUCCUGUUCCUGUCCCA*
	PB1-5	GAUUUAGACUACCCCAAAAACGAAGGGGACUAAAAC *CUGUUGCAGCAGGCUGGUUUCUAUUUAA*
	PB1-6	GAUUUAGACUACCCCAAAAACGAAGGGGACUAAAAC *GACUCCAUUACAUCCUUAAGGAAGUCUA*
NP	NP-1	GAUUUAGACUACCCCAAAAACGAAGGGGACUAAAAC *AUUUCCCUUUGAGAAUGUUGCACAUUCU*
	NP-2	GAUUUAGACUACCCCAAAAACGAAGGGGACUAAAAC *UUUUCGUCCAUUCUCACCCCUCCAGAAG*
	NP-3	GAUUUAGACUACCCCAAAAACGAAGGGGACUAAAAC *UCCAUCAUUGCUUUUUGUGCAGCAGUUU*
	NP-4	GAUUUAGACUACCCCAAAAACGAAGGGGACUAAAAC *UCUCCGAAGAAAUAAGAUCCUUCAUUAC*
	NP-5	GAUUUAGACUACCCCAAAAACGAAGGGGACUAAAAC *GGCAGGCAGGACUUGUGAGCAACCGACC*
	NP-6	GAUUUAGACUACCCCAAAAACGAAGGGGACUAAAAC *UUGCACUUUCCAUCAUCCUUAUGAUUUC*
PB2	PB2-1	GAUUUAGACUACCCCAAAAACGAAGGGGACUAAAAC *GAUAUUUCAUUGCCAUCAUCCAUUUCAU*
	PB2-2	GAUUUAGACUACCCCAAAAACGAAGGGGACUAAAAC *AAAGUUUGUCCUUGCUCAUUUCUCUCAG*
	PB2-3	GAUUUAGACUACCCCAAAAACGAAGGGGACUAAAAC *UCCUAUUCCACCAUGUCACAGCCAGAGG*
	PB2-4	GAUUUAGACUACCCCAAAAACGAAGGGGACUAAAAC *AGUUUUGUAGAUUUUUGGAUAAUGAACU*
	PB2-5	GAUUUAGACUACCCCAAAAACGAAGGGGACUAAAAC *CAGGGCCAAAGGUUCCAUGCUUUAGCCU*
	PB2-6	GAUUUAGACUACCCCAAAAACGAAGGGGACUAAAAC *UCCGACGUAUUUUGACUUGGUUUCUAAA*
NS	NS-1	GAUUUAGACUACCCCAAAAACGAAGGGGACUAAAAC *AUUGCUCCCUCUUCGGUGAAAGCCCUUA*
	NS-2	GAUUUAGACUACCCCAAAAACGAAGGGGACUAAAAC *AUGUCCUGGAAGAGAAGGCAAUGGUGAA*
	NS-3	GAUUUAGACUACCCCAAAAACGAAGGGGACUAAAAC *UUUCAGAGACUCGAACUGUGUUAUCAUU*
	NS-4	GAUUUAGACUACCCCAAAAACGAAGGGGACUAAAAC *CAAACUUCUGACCUAAUUGUUCCCGCCA*
	NS-5	GAUUUAGACUACCCCAAAAACGAAGGGGACUAAAAC *AGUGCCUCAUCGGAUUCUUCUUUCAGAA*

### RT-PCR-CRISPR detection methods

The RT-PCR amplified template was the RNA of the influenza A PR8 strain. The primers were listed in **Table 2**. The reaction system was as follows: 2×ExBuffer, 12.5μL; RT-Enzyme, 0.5μL; ExTaq, 0.5μL; Forward primer, 1μL; Reverse primer, 1μL; RNA, 2μL; RNase-free H_2_O, 7.5μL. The reaction procedure is as follows: 37°C, 15min, 1cycle; 85°C, 5min, 1cycle; 95°C, 30s, 95°C, 10s, 55°C, 30 s, 40cycle; 72°C, 30s, 1cycle; 72°C, 10min,1cycle; 4°C, ∞. For CRISPR detection, The reaction system was as follows: 1.6 IU/μL RNase inhibitor (NEB), 20 mM N-2-hydroxyethyl piperazine-N-2-ethane sulfonic acid (HEPES, NEB), 25 nM Cas13a (Genscript), 2 μM crRNA, 2.5 mM ribonucleoside triphosphates (NEB), 2 nM reporter RNA (Invitrogen), 1 IU/μL T7 RNA polymerase (NEB), 10 mM MgCl_2_, and 5 μL of the RT-PCR amplified product. The reaction was incubated and detected for 60 min at 37°C using the fluorescence quantitative PCR instrument MasterCycler RealPlex4 (Eppendorf).

**Table 2:** RT-PCR primers sequence in our study.

name	Sequence (5’→3’)
PA-F	taatacgactcactatagGCAGGTACTGATCCAAAATG
PA-R	CCATCCACATAGGCTCTAAA
PB1-F	taatacgactcactatagCCCTTATACCGGAGACCCTC
PB1-R	CTGGCAACCCTGATTGTTCA
NP-F	taatacgactcactatagGATGGAATTGGTCAGGATGA
NP-R	TAATTGTCGTACTCCTCTGC
PB2-F	taatacgactcactatagGACAGGAGAAGAACCCAGCA
PB2-R	GAGATCTGCATGACCAGGAT
NS-F	taatacgactcactatagCGTGCTGGAAAGCAGATAGT
NS-R	CTCTTGCTCCACTTCAAGCA

Lowercase is T7 transcript recognition sequences.

### Cell lines and viral culture

A549 and Madin-Darby canine kidney (MDCK) cells were grown in Dulbecco’s modified Eagle medium (DMEM) supplemented with 10% heat-inactivated fetal bovine serum, 1% penicillin and streptomycin, and 100 mg/mL L-glutamine. A/Puerto Rico/8/1934(H1N1) (provided by the Center for Disease Control and Prevention of the Chinese People’s Liberation Army) viral culture supernatant was harvested in minimal Eagle medium (MEM) from MDCK cells, filtered through a 0.2 mm filter, titrated by a plaque test on MDCK cells, and stored at -80°C.

### Viral infection and transfection

During virus adsorption, crRNA/Cas13a complexes were prepared according to optimized conditions: 500 ng Cas13a protein, 125 ng crRNA, 1 μL Reagent PLUS, and 25 μL Opti-MEM in tube 1, and 1.5 μL Lipofectamine CRISPR MAX reagent (Thermo) and 25 μL Opti-MEM in tube 2. The components were mixed thoroughly and incubated for 5 min at room temperature, after which those of both tubes were mixed and incubated at RT for 25 min. The complexes containing crRNA-Cas13a, Cas13a alone, or the transfection reagent alone were added to PR8-infected cells and incubated for 4 h. Finally, 0.5 mL of DMEM containing 1% bovine serum albumin (BSA), 1% penicillin and streptomycin, glutamine, and 1 mg/mL L-1-tosylamido-2-phenylethyl chloromethyl ketone (TPCK) were added to each well and incubated at 37°C and 5% CO_2_ for 24 h. Cells were harvested to extract RNA and viral RNA was detected by reverse transcription-quantitative polymerase chain reaction (RT-qPCR). Infectious viral particles were verified by median tissue culture infectious dose (TCID50), and plaque assays.

### RT-qPCR assay

Viral RNA was extracted from cell particles using a QIAamp Viral RNA Mini kit (Qiagen), after which viral RNA was quantified using a one-step TB Green PrimeScript RT-PCR kit (TAKARA). A universal primer pair for detecting the Matrix protein (M) segment of the influenza A virus was used, the forward primer was 5’-TTCTAACCGAGGTCGAAACG-3’, the reverse primer was 5’-ACAAAGCGTCTACGCTGCAG-3’. β-actin (forward primer: 5’-CCTGGCACCCAGCACAA-3’; reverse primer: 5’-GCCGATCCACACGGAGTACT-3’) was used as an internal control. The reactions were performed as follows: reverse transcription at 42°C for 5 min, denaturation at 95°C for 10 s, and 40 cycles of amplification at 95°C for 5 s and 60°C for 30 s.

### Plaque assay

Different experimental samples were diluted 10 times and then added to MDCK cells in 12-well plates. Then they were incubated at 37°C and 5% CO_2_ for 90 min to allow virus adhesion, dissolved with 1.5% methylcellulose, mixed with 2× DMEM in an equal proportion, and covered with 1% BSA and 2 mg/mL TPCK pancreatic enzyme. The cells were incubated at 37°C and 5% CO_2_ for 3 days and fixed with 4% paraformaldehyde at room temperature for 30 min. After washing with water, 2% crystal violet dye was added to the cells, then incubated at room temperature for 30 mins. After washing with water, plaques were counted, and the viral titer was expressed as a plaque-forming unit (PFU)/mL.

### TCID50 assay

After 10-fold gradient dilution, different experimental samples were added to MDCK cells in 96-well plates and incubated at 37°C and 5% CO_2_ for 90 min to allow the virus adhesion. After discarding the diseased venom, DMEM containing 1% BSA and 2 mg/mL TPCK trypsin was added to each well. The cells were incubated at 37°C and 5% CO_2_ for 3 days, and cytopathy was observed daily. The viral titer was calculated after the cells stopped exhibiting pathological changes, and the viral titer was represented as IogTCID50.

### Statistical analysis

Statistical significance was determined by unpaired two-tailed Student’s *t*-test using GraphPad Prism 8.0.2 software. Data are presented as the mean ± standard error of the mean. The levels of significance were indicated as follows: n.s., no significant difference; *p < 0.05, **p < 0.01, ***p < 0.001, and ****p < 0.0001.

## Results

### CrRNA screening using CRISPR-Cas13a-based nucleic acid detection

**Figure 1:**
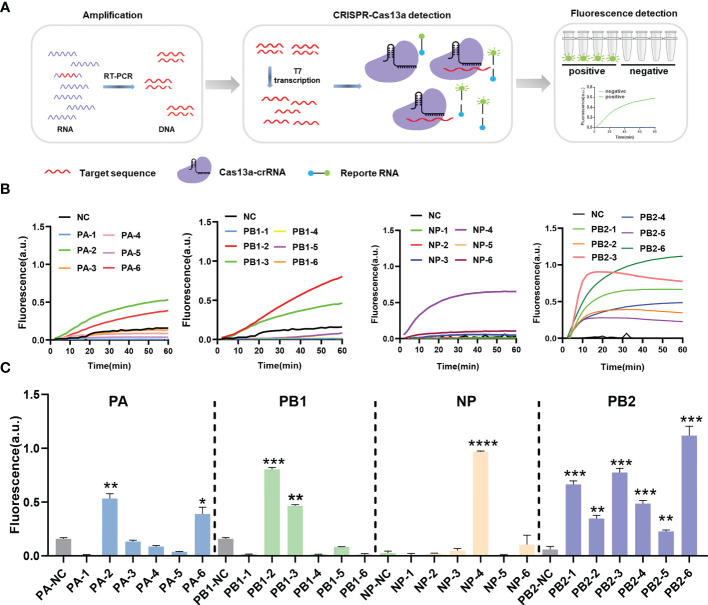
CrRNA screening using the CRISPR-Cas13a nucleic acid detection system. **(A)** Schematic of reverse transcription polymerase chain reaction-CRISPR detection. **(B)** Results of screening different crRNAs. The fluorescence values of crRNA at different times. n=3 replicates. **(C)** The fluorescence values of different crRNAs at 60 min. n=3 replicates. Data are analyzed by *t*-test. Bars represent the mean ± standard error of the mean SEM. * *p* < 0.05; ** *p* < 0.01; ***p < 0.001; ****p < 0.0001.

To avoid escaping mutant viruses, the conserved regions of the PA, PB1, NP, and PB2 segments were screened *via* sequence alignment. Six crRNAs were designed for each segment (**Table 1**). The detection efficiency of 24 crRNAs was evaluated using RT-PCR-CRISPR detection (**Figure 1A**). The results showed that 11 of the 24 crRNAs in four segments could effectively target RNA: PA-2, PA-6, PB1-2, PB1-3, NP-4, PB2-1, PB2-2, PB2-3, PB2-4, PB2-5, PB2-6 (**Figures 1B, C**). In the PA segment, the highest fluorescence value of PA-2 was 0.5737 ± 0.0523 a.u., and the highest fluorescence value of PA-6 was 0.4329 ± 0.0390 a.u. (**Figures 1B, C**). In the PB1 segment, the highest fluorescence value of PB1-2 was 0.9412 ± 0.0076 a.u., and the highest fluorescence value of PB1-3 was 0.5230 ± 0.0102 a.u. (**Figures 1B, C**). In the NP segment, the highest fluorescence value of NP-4 was 0.9666 ± 0.0079 a.u. (**Figures 1B, C**). In the PB2-1 segment, the highest fluorescence value of six crRNAs was 0.6643± 0.0324, 0.3468 ± 0.0305, 0.7747 ± 0.0387, 0.4862 ± 0.0279, 0.2265 ± 0.0142, 1.1186 ± 0.0855 respectively (**Figures 1B, C**). Overall, our results suggested that the CRISPR-Cas13a system containing the above crRNAs had a high cleavage activity.

### Evaluation of the antiviral effect of crRNAs screened by CRISPR-Cas13a-based nucleic acid detection

**Figure 2:**
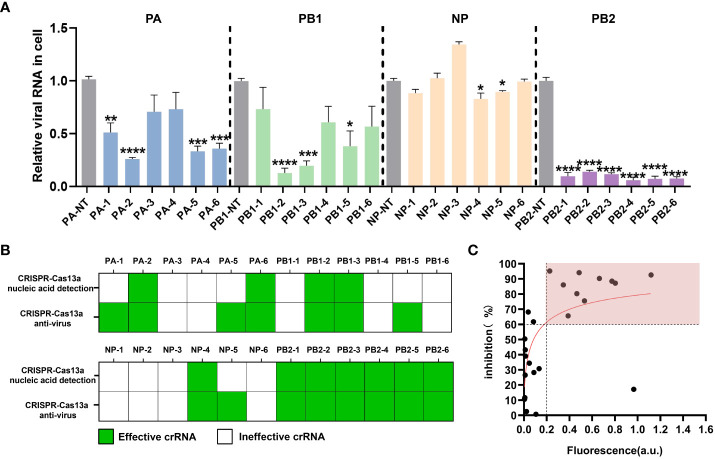
Evaluation of the antiviral effect of the CRISPR-Cas13a system. **(A)** Inhibitory efficacy of different crRNAs, results are expressed as the ratio of the viral RNA copy number of each treatment to that of the untargeted crRNA group, which was set to 1. n=3 replicates. Data are analyzed by *t*-test. Bars represent the mean ± SEM. * *p* < 0.05; ** *p* < 0.01; *** *p* < 0.001;**** *p* < 0.0001. **(B)** the statistical results of effective crRNAs (green) and ineffective crRNAs (white) screened by CRISPR-Cas13a fluorescence detection and verified by RT-qPCR. **(C)** Correlation between the maximum fluorescence value of crRNA in CRISPR-Cas13a fluorescence detection and the inhibition rate of viral load in cells.

To explore the inhibitory effect of the CRISPR-Cas13a system containing different crRNAs on PR8 viral load in cell samples, we synthesized crRNAs (**Table 1**) and prepared crRNA/Cas13a complexes for transfection. RT-qPCR was used to quantify the copy number of viral RNA in the cells to evaluate antiviral activity. The results showed that 15 of the 24 crRNAs could reduce viral load, including 11 crRNAs screened by CRISPR-Cas13a nucleic acid detection. In the PA segment, compared with the non-targeting crRNA (NT-crRNA) group, the inhibition rates of the CRISPR-Cas13a systems containing PA-2 and PA-6 on viral load were 75.52 ± 3.21% and 65.63 ± 5.89% respectively (**Figure 2A**). In the PB1 segment, the inhibition rates of the CRISPR-Cas13a systems containing PB1-2 and PB1-3 on viral load were 87.20 ± 5.33% and 80.26 ± 5.29% respectively (**Figure 2A**). In the NP segment, the CRISPR-Cas13a systems containing NP-4 could decrease the viral load by 17.15 ± 6.03% (**Figure 2A**). In the PB2 segment, the CRISPR-Cas13a systems containing PB2-1, PB2-2, PB2-3, PB2-4, PB2-5, PB2-6 could decrease viral load by 90.28% ± 4.71%, 86.07% ± 3.47%, 88.46% ± 3.47%, 94.1% ± 4.3%, 95.18% ± 4.24%, 92.6 ± 3.65% respectively (**Figure 2A**). Based on the above results, we verified the effective antiviral activity of crRNAs screened by CRISPR-Cas13a nucleic acid detection.

### Establishment of an efficient crRNA screening platform for anti-influenza virus

Based on the antiviral efficiency of the crRNAs screened by CRISPR-Cas13a nucleic acid detection, we explore the feasibility of the anti-influenza virus crRNA screening platform. We analyzed effective crRNAs screened by CRISPR-Cas13a nucleic acid detection and CRISPR-Cas13a antivirus in cells. For CRISPR-Cas13a nucleic acid detection, the effective crRNA means that the fluorescence value is significantly greater than that of the control group. For the CRISPR-Cas13a antivirus, the effective crRNA means that the virus load verified by RT-qPCR is significantly lower than that of the control group. Although four crRNA, PA-1, PA-5, PB1-5, and NP-5, were not effective when screened by CRISPR-Cas13a nucleic acid detection, they were verified to be effective by RT-qPCR in cells. The 11 crRNAs, PA-2, PA-6, PB1-2, PB1-3, NP-4, PB2-1, PB2-2, PB2-3, PB2-4, PB2-5, PB2-6, screened by CRISPR-Cas13a nucleic acid detection, could be effective for antiviruses in cells. Thus, the antiviral effectiveness of crRNAs screened by CRISPR-Cas13a nucleic acid detection was 100% (**Figure 2B**). We further found that the fluorescence values showed a logarithmic relationship with viral inhibition rate. For 91% (10/11) of crRNAs, when the maximum fluorescence value of CRISPR-Cas13a nucleic acid detection was greater than 0.2 a.u., the viral inhibition rate was greater than 60% (**Figure 2C**). Based on these results, we initially established an anti-influenza virus crRNA screening platform based on CRISPR-Cas13a nucleic acid detection.

**Figure 3:**
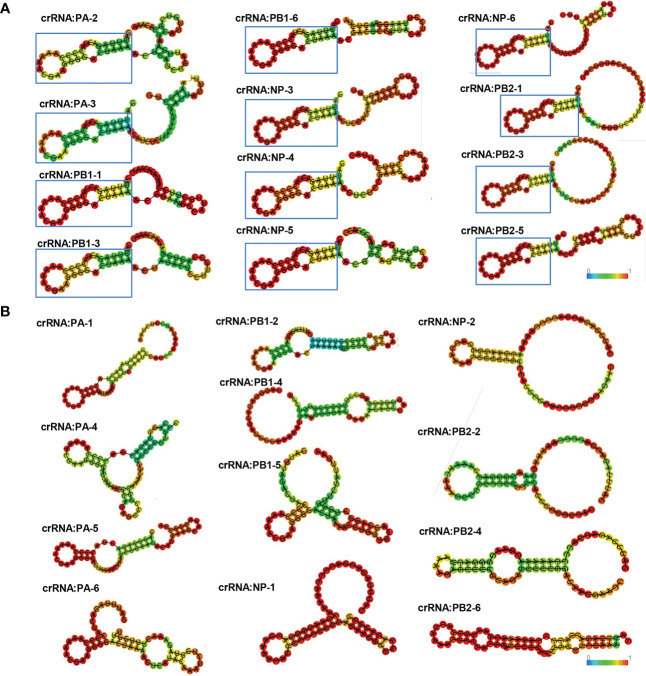
crRNA secondary structure prediction. **(A)** Effective crRNAs of PA, PB1, NP, and PB2, predicted by secondary structure prediction. The crRNA scaffold forms a stable secondary structure containing a stem-loop to bind to Cas13. Colors represent base-pair probabilities. **(B)** Ineffective crRNAs of PA, PB1, NP, and PB2, predicted by secondary structure prediction.

The RNase activity of Cas13a is related to the secondary structure of crRNA. RNA secondary structure prediction by bioinformatics has been used to screen antiviral targets. To compare this platform with the method of RNA secondary structure prediction, we predicted and analyzed the secondary structure of a total of 24 crRNAs of PA, PB1, NP, and PB2. According to previous studies, the structure of crRNA includes scaffold and guide sequences. The crRNA scaffold forms a stable secondary structure to bind Cas13 protein and guide sequences bind target RNA. The results of secondary structure prediction showed that crRNAs namely PA-2, PA-3, PB1-1, PB1-3, PB1-6, NP-3, NP-4, NP-5, NP-6, PB2-1, PB2-3 and PB2-5 had stable hairpin structures that could bind to Cas13a protein (**Figures 3A, B**). However, PA-3, PB1-1, PB1-6, NP-3, and NP-6 were verified to have no antiviral effects in cells. Thus, the results indicate that CRISPR-Cas13a nucleic acid detection is more accurate than crRNA secondary structure prediction.

### Application and evaluation of the antiviral crRNA screening platform

**Figure 4:**
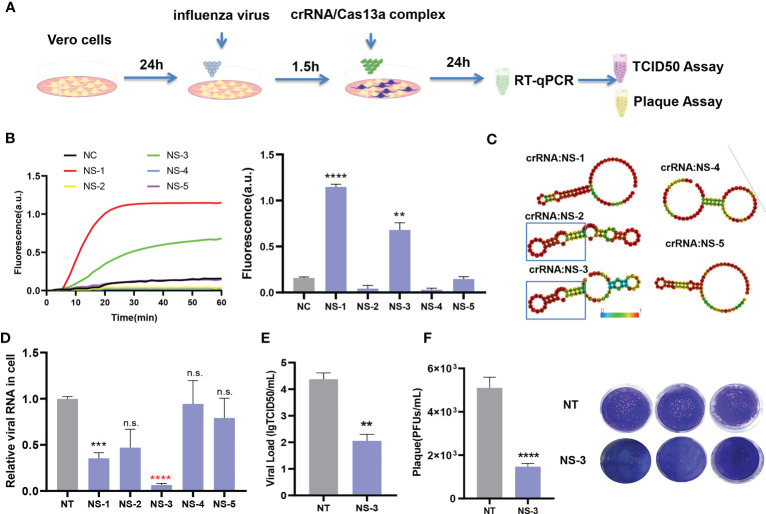
Application and evaluation of crRNA screening platform. **(A)** Schematic diagram of the assessment process. **(B)** Detection results of NS crRNAs by CRISPR-Cas13a fluorescence detection. n=3 replicates. Data are analyzed by *t*-test. Bars represent the mean ± SEM. ** *p* < 0.01;*** *p* < 0.001. **(C)** Prediction results of NS crRNA secondary structure. **(D)** Inhibition rate of viral load by CRISPR-Cas13a systems containing different crRNAs. n=3 replicates. Data are analyzed by *t*-test. Bars represent the mean ± SEM. *** *p* < 0.001;**** *p* < 0.0001; n.s. represents no significant difference. The red star indicates common crRNA screened by CRISPR-Cas13a fluorescence detection and secondary structure prediction. **(E)** TCID50 detection of NS-3 crRNA group. The results are expressed as logTCID50. n=3 replicates. Data are analyzed by *t*-test. Bars represent the mean ± SEM. ** *p* < 0.01. **(F)** The average number of infected virions was quantified by plaque assay and plaque image. n=3 replicates. Data are analyzed by *t*-test, Bars represent the mean ± SEM, ****, *p* < 0.0001.

To further evaluate this platform, the crRNAs targeting the NS segment were screened, and their antivirus efficiency (**Figure 4A**). A conserved region in the NS segment of the influenza A (H1N1) virus was used to design the crRNAs (**Table 1**). Five crRNAs were screened using nucleic acid detection, meantime RNA secondary structures were predicted. The results showed that the CRISPR-Cas13a system containing NS-1 and NS-3 could be detected the fluorescence signals (**Figure 4B**). The highest fluorescence value of NS-1 was 1.1477 ± 0.0293 a.u., and the highest fluorescence value of NS-3 was 0.6805 ± 0.0771 a.u. We analyzed the secondary structure of the crRNAs to predict their antiviral ability. NS-2 and NS-3 were found to have a stable hairpin structure (**Figure 4C**). We then assessed the antiviral activity by RT-qPCR. The CRISPR-Cas13a systems containing NS-1 and NS-3 could effectively inhibit the virus, same as the results of CRISPR-Cas13a nucleic acid detection. The viral load decreased by 68.23 ± 7.49% and 94.27 ± 2.19% respectively (**Figure 4D**). The results again indicate that our platform is more accurate than RNA structure prediction. The NS-3 was the common crRNA screened by CRISPR-Cas13a nucleic acid detection and RNA secondary structure prediction, It had the highest inhibition efficiency. Then the effectiveness of the NS-3 was verified by TCID50 assay and plaque assay. The TCID50 assay results showed that compared with the NT-crRNA group, the viral titer of the NS-3 treatment group was reduced by 99.52 ± 1.87% (**Figure 4E**). In addition, plaque assay results showed that compared with the NT-crRNA group, the viral PFU of the NS-3 treatment group was reduced by 71.24 ± 7.05% (**Figure 4F**). The above results show that the CRISPR-Cas13a system containing NS-3 can efficiently inhibit infectious viral particles. Therefore, our platform could quickly and accurately screen antivirus crRNA, and also could combine with the secondary structure prediction of crRNA to improve the efficiency of screening crRNA.

## Discussion

CRISPR technology has been widely used in agronomy (20, 21), biology (22, 23), and medicine (24, 25) owing to its efficient programmability. In recent years, the CRISPR-Cas13a system has shown great potential as a new antiviral technology. Some studies have shown that the CRISPR-Cas13 system can effectively inhibit RNA viruses, such as lymphocytic choriomeningitis, vesicular stomatitis, dengue, and severe acute respiratory syndrome-associated virus 2. Compared with other antiviral technologies such as RNA interference, the CRISPR-Cas13 system has lower off-target effects and higher efficiency (18). Previous studies have shown the application prospect of the CRISPR-Cas13 system as an efficient programmable antiviral platform. Rapid screening of antiviral crRNA targets is key for the CRISPR-Cas13a system to achieve high antiviral efficiency. In this study, we established the CRISPR-Cas13a nucleic acid detection based on the same principle as the published articles. And nucleic acid detection and antiviral technology based on the CRISPR-Cas13a system were combined for the first time to establish an efficient crRNA-screening technique. Subsequently, this technology was used to efficiently inhibit influenza A (H1N1) viruses.

We established the antiviral crRNA screening platform based on CRISPR-Cas13a nucleic acid detection by targeting PA1, PB1, NP, and PB2. CRISPR-Cas13a system can effectively inhibit the virus by targeting the genes encoding the PA, PB1, NP, and PB2 of the influenza virus. RNA-dependent RNA polymerase (RdRp), which is composed of PA, PB1, and PB2 proteins, binds to and enters the nucleus to initiate replication and transcription, forming mRNA for the production of other viral proteins. PA, PB1, and PB2 are critical for IAV replication and transcription (26, 27). NP is a highly conserved and multifunctional protein. NP facilitates the import and export of viral ribonucleoprotein complexes from the nucleus and is involved in the selective packaging of viral gene fragments (28, 29). As a non-structural protein, the NS protein can prevent the nuclear export of mRNA from host cells and promote RNA replication and protein expression by cutting the polyadenylate cap of the host mRNA as a primer for viral RNA synthesis (30, 31). Therefore, we speculated that when the CRISPR-Cas13 system targets PA, PB1, NP, PB2, and NS segments, it inhibits the transcription and replication of viral RNA and affects the viral life cycle, thus exerting a highly effective antiviral effect. Notably, the antiviral effect of the CRISPR-Cas13a system may be affected by several factors. First, different gene segments targeted by the CRISPR system have different inhibitory effects on the virus, which may be related to whether the gene plays a key role in the viral life cycle, suggesting that key genes affecting viral replication can be selected as targets.

The secondary structure of crRNA could affect the antiviral effect of the CRISPR-Cas13 system. The binding of crRNA to target RNA can affect the cleavage activity of the CRISPR system (32). Meanwhile, whether crRNA can bind to Cas13a protein could affect the formation of the crRNA-Cas13a complex (33). The crRNA forms a specific secondary structure that binds to the Cas13a protein to activate Cas13a cleavage activity. Previous studies showed that the crRNA scaffold forms a bulge-containing stem-loop structure to bind Cas13a (11). In this study, we predicted the secondary structure of crRNA targeting PA1, PB1, NP, PB2, and NS to screen antiviral crRNA. The results suggested that several crRNAs screened by RNA secondary structure prediction could not be used for effective antiviral. However, the effectiveness of crRNA screened by our platform is 100%. Therefore, our method is more sensitive.

In conclusion, this study is the first to combine the CRISPR-Cas13a nucleic acid detection and antiviral technologies to establish a high-efficiency anti-influenza virus crRNA target screening technology, and it demonstrated the feasibility of this technology through further research. The platform allows for efficient, rapid, high-throughput, and low-cost screening against influenza virus crRNA targets, shortens the screening cycle of CRISPR antiviral targets, and provides a powerful tool for promoting the application and development of programmable antiviral technologies based on the CRISPR-Cas13 system.

## Data availability statement

The original contributions presented in the study are included in the article. Further inquiries can be directed to the corresponding authors.

## Author contributions

YSS, HL, YZ, and LY conceived the experiments. YZ, WY, XD, and MN performed the experiments. LY performed the RNA structure prediction. YZ and YH conducted statistical analysis. LY, YZ, and WY prepared the tables and figures. LY, HL, YZ, and YJS prepared the manuscript, LY and HL revised the manuscript. All authors have reviewed and approved the final manuscript.
